# Enhancement of Charge Transfer and Quenching of Photoluminescence of Capped CdS Quantum Dots

**DOI:** 10.1038/srep12056

**Published:** 2015-07-13

**Authors:** Mohan Singh Mehata

**Affiliations:** 1 Laser-Spectroscopy Laboratory, Department of Applied Physics, Delhi Technological University, Bawana Road, Delhi 110042, INDIA

## Abstract

Quantum dots (Q-dots) of cadmium sulfide (CdS) with three different capping ligands, 1-butanethiol (BT), 2-mercaptoethanol (ME) and benzyl mercaptan (BM) have been investigated. An external electric field of variable strength of 0.2–1.0 MV cm^−1^ was applied to the sample of capped CdS Q-dots doped in a poly(methyl methacrylate) (PMMA) films. Field-induced changes in optical absorption of capped CdS Q-dots were observed in terms of purely the second-derivative of the absorption spectrum (the Stark shift), indicating an enhancement in electric dipole moment following transition to the first exciton state. The enhancement depends on the shape and size of the Q-dots prepared using different capping ligands. Field induced-change in photoluminescence (PL) reveals similar changes, an enhancement in charge-transfer (CT) character in exciton state. PL of capped CdS Q-dots is significantly quenched in presence of external electric field. The strong field-induced quenching occurs as a result of the increased charge separation resulting exciton dissociation. Thus, understanding the CT character and field-induced PL quenching of CdS Q-dots is important for photovoltaic, LEDs and biological applications.

Nanometer-sized semiconductor materials have been the subject of great interest in the field of physics, chemistry and biology for fundamental research and their potential applications^1^,^2^,^3^,^4^,^5^,^6^,^7^. In the recent past, quantum dots (Q-dots) with their unique properties have attracted much attention^1^,^2^,^3^. Cadmium sulfide (CdS) Q-dots, a semiconductor is known for the size-dependent optical absorption and photoluminescence (PL) in ultraviolet-visible region, and the dimension of CdS Q-dots is matched with biological molecules^4^,^5^,^6^,^7^,^8^. The spectroscopic tool exhibits a clear quantum size effects in optical properties in term of change in color, spectral shifts to the higher energy side with the reduction of particle size, and also in term of enhanced oscillator strength^5^,^9^. In fact, the size and structure dependent properties make them suitable for the potential applications of light generation or fluorescent biological labeling, optoelectronic devices, solar energy conversion etc.^10^,^11^,^12^. Several methods, including UV and microwave irradiation (MW) were used for the preparation of CdS nanoparticles. The MW assisted synthesis is important because it requires short time to produce Q-dots with a narrow particle size distribution^6^,^7^,^8^.

The photophysical properties of CdS nanoparticles have been examined with steady-state and time-resolved techniques^4^,^5^,^6^,^7^,^8^. Typically, CdS nanoparticles exhibits dual emission in the visible region^5^,^12^. The emission band appeared at higher energy sites ascribed to a direct recombination of *electron-hole* pair at the band gap, whereas the lower energy emission originates due to radiative recombination at deep trap sites originating from surface states, i.e., lattice imperfections at the surface^5^,^12^. Especially, thiol capped CdS nanocrystallites shows single emission band^6^, which has been considered to be originated from the surface vacancy/defects. Thus, the study of CdS nanocrystallites prepared by using different capping ligands opened new avenues to control the size and structure of CdS nanoparticles, which further produces a significant modification in spectroscopic properties. Furthermore, application of an external electric field plays a decisive role in understanding electronic structure and excited-state dynamics of the small size such as nanometer size materials Q-dots as well as large molecules such as polymers and protein^5^,^7^,^13^,^14^,^15^,^16^,^17^,^18^,^19^,^20^,^21^.

The application of an external field to the optical transitions provides important information about the charge-transfer reaction and molecular motion^13^,^14^,^15^. This also provides information about the reliability and stability of organic light emitting diode (OLEDs)^20^,^21^ or polymer light emitting diode (PLED)^16^,^17^, and more efficiently accelerated and decelerated electron transfer processes in molecular systems^14^. It has been observed that the PL of CdS and cadmium telluride (CdTe) nonoparticles is quenched by the application of external electric field, indicating that applied electric field influence the excited-state dynamics of nanomaterials^5^,^7^,^18^. The electric field modulation and quantum confinement Stark effect of CdSe nanoparticles exhibits dipolar character both in the ground and excited states, i.e. CdSe nanoparticle possess ground state dipole moment^3^,^19^. Such a dipolar character is also observed for the CdS nanoparticles, and found to be more prominence in capped nanoparticles^5^,^7^.

In previous studies^5^,^7^, the field-induced change in absorption, emission and emission decay of uncapped and benzyl mercaptan (BM) capped CdS Q-dots doped in a PMMA films were reported. In which the uncapped Q-dots shows dual emission whereas BM capped CdS Q-dots shows single large Stake’s shifted as well as stable emission. Keeping in view the unusual photophysical behavior of CdS Q-dots with/without capping, the study is further explored. To control the shape and size of CdS nanoparticles, various capping ligands, that is, 1-butanethiol (BT), 2-mercaptoethanol (ME) and BM were used. Then capped CdS Q-dots were doped in a poly(methyl methacrylate) (PMMA) films for the application of simultaneous fields, light (an electromagnetic) and electric field (static field) and a comparative study were presented. Electroabsorption (E-A) and electro-photoluminescence (E-PL) spectra of capped CdS Q-dots were measured by recording plots of the field-induced changes in absorption and photoluminescence intensity as a function of wavelength or wavenumber at twice of the frequency (2ω) of the applied electric field. E-PL spectra were measured at varying electric field strength and excitations. Based on the results, electronic structure and charge transfer dynamics following optical transitions have been examined for the capped CdS Q-dots.

## Results and Discussion

Electric field effects on spectral changes were described in the recent past^13^,^14^,^15^,^16^,^17^ starting from Liptay’s the Stark effect theory^22^. A shift of energy level induced by applied electric field is usually known as the Stark effect, and depends on the electric dipole moment (μ) and the polarizability (α) of the states of the system concerned. When the magnitude of ***μ*** and ***α*** in the excited electronic state is different from that in the ground state, the optical absorption and photoluminescence spectra are shifted towards lower/higher energy sites since the magnitude of the level shift in both states are different from each other.

On the assumption that the original isotropic distribution of CdS Q-dots in a PMMA film is maintained in the presence of electric field, the field-induced change in absorption intensity (i.e., E-A spectrum) at wavenumber *ν,* ΔΑ(ν) may be given by a linear combination of the absorption its first and second derivative spectra as follows^13^,^14^,^15^,^16^,^17^:
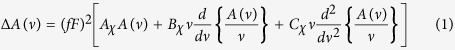
Similarly, the field induced change in PL intensity (i.e., E-PL spectrum) can also be given as:
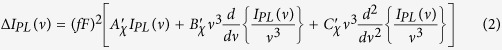
in which *f* and *F* (=|***F***|) represent the internal field factor and magnitude of applied electric field, respectively. In an immobilized and randomly distributed system, the zeroth derivative coefficient *A*_*χ*_ and
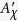
originates from the change in transition moment induced by electric fields and the field-induced change in emission intensity, respectively. The first derivative coefficient *B*_*χ*_ (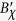) and second derivative coefficient *C*_*χ*_ (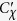) correspond to the spectral shift and spectral broadening of the E-A and E-PL spectra resulting from the change in polarizabilty (*Δα*) and electric dipole moment (*Δμ*), respectively in the excited state with respect to the ground state. The coefficients *B*_*χ*_ (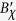) and C_*χ*_ (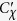) are given by the following equations:
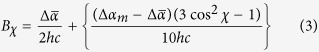

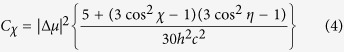
where Δμ and Δα are the differences in electric dipole moment and polarizability, respectively between the ground state (*g*) and excited state (*e*), i.e., 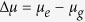
and 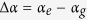. 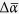 denotes the trace of 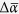, i.e., 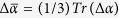, Δ*α*_*m*_ represents the diagonal component of 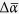 with respect to the direction of the transition dipole moment; *η* is the angle between the direction of Δμ and the transition dipole moment; *χ* is the angle between the direction of applied electric field and the electric vector of the incident light used for the measurement of E-A spectra. At magic angle condition of *χ* ( = 54.7^°^), the first and second derivative coefficients (*B*_*χ*_ and *C*_*χ*_) are given by the following equations:
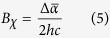

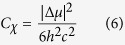
From equations 1, 2, 3, 4, 5, 6, the values of 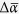 and 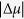 can be obtained from the analysis of the first and second derivative of the E-A and E-PL spectra, respectively.

**Figure 1:**
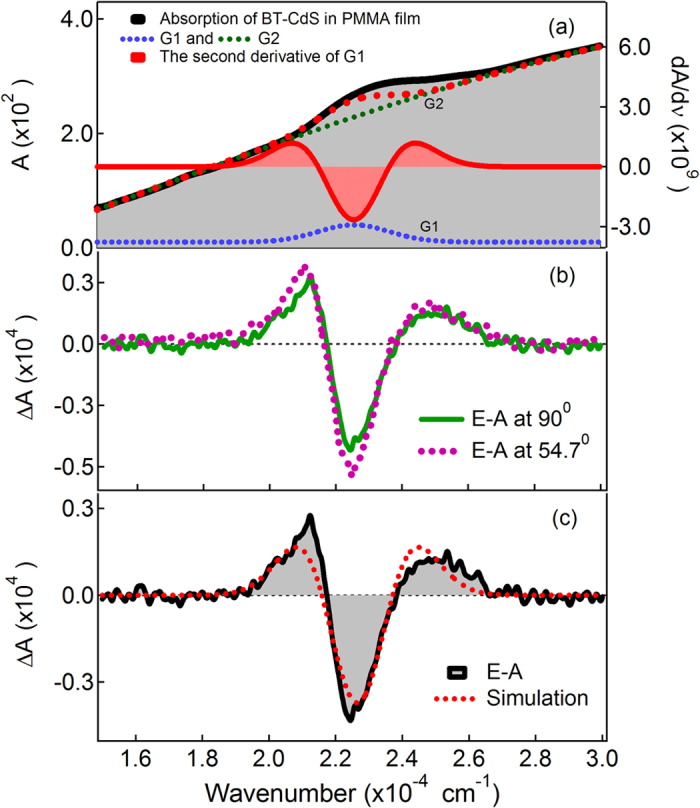
(**a**) Absorption spectrum with a Gaussian functions and the second derivative spectrum of G1 Gaussian function, (**b**) electroabsorption (E-A) spectra of BT-capped CdS Q-dots embedded in a PMMA film observed at two different angles of *χ* (=90° and 54.7°) at field strength of 0.7 MV cm^−1^ at 295 K. (**c**) E-A spectrum with the simulated spectrum.

**Figure 2:**
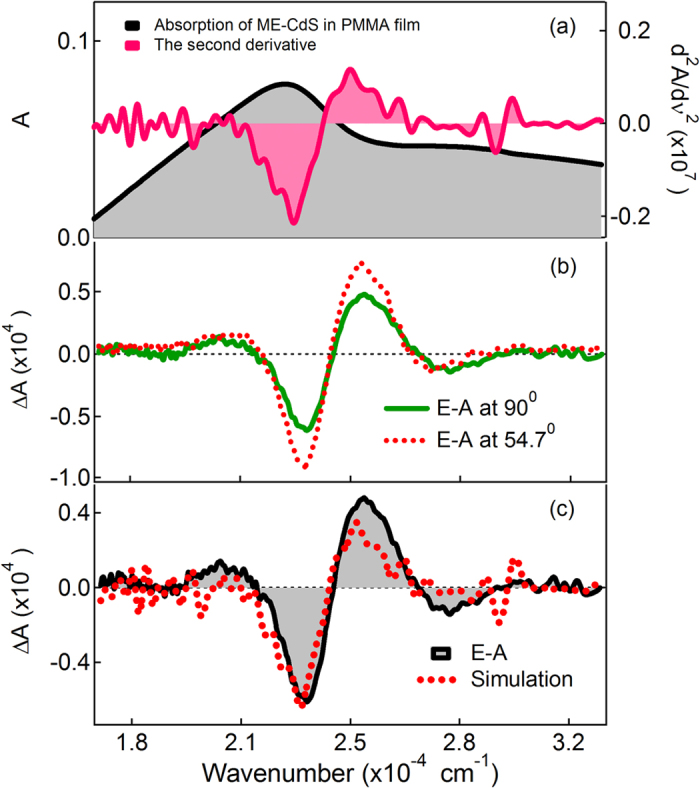
(**a**) Absorption spectrum and the second derivative of the absorption spectrum and (**b**) electroabsorption (E-A) spectra of ME-capped CdS Q-dots embedded in a PMMA film observed at two different angles of *χ* (=90° and 54.7°) at field strength of 0.7 MV cm^−1^ at 295 K. (**c**) E-A spectrum with the simulated spectrum.

**Figure 3:**
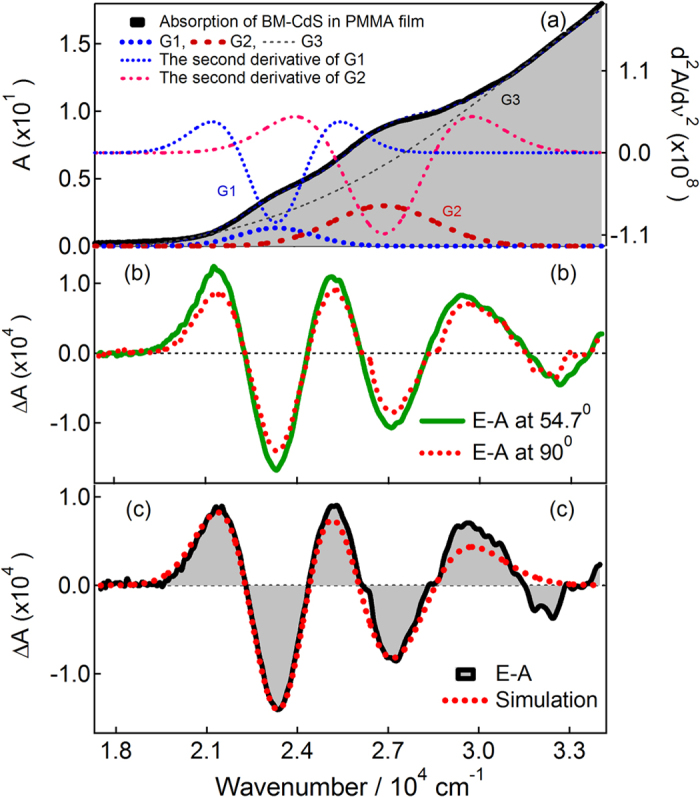
(**a**) Absorption spectrum with Gaussian functions together with the second derivative spectrum of G_1_ and G_2_ Gaussian functions, (**b**) electroabsorption (E-A) spectra of BM-capped CdS Q-dots embedded in a PMMA film observed at two different angles of *χ* ( = 90^°^ and 54.7^°^) at a field strength of 0.7 MV cm^−1^ at 295 K. (**c**) E-A spectrum with the simulated spectrum^7^.

Figures 1, 2, 3 show the electroabsorption (E-A) and absorption spectra of capped CdS Q-dots doped in a PMMA film. These spectra were recorded in a range of 15000–30000 cm^−1^ for BT capped and 16500–32500 cm^−1^ for ME and 17500–34000 cm^−1^ for BM capped CdS Q-dots. The average size of the Q-dots estimated from the absorption spectra is 3.8, 4.0 and 2.1 nm for BT, ME and BM capped CdS Q-dots, respectively. The absorption spectrum of BT and BM capped Q-dots was reproduced by using Gaussian functions, which are used in the simulation of the E-A spectra, except for ME capped Q-dots. In each case, the lowest energy absorption band is corresponding to the transitions to the first exciton state, which represents to the direct band gap of CdS Q-dots. The energy band gap of the capped CdS Q-dots varies with the particle size. A single absorption maximum is observed at 442 and 444 nm respectively for BT and ME capped Q-dots, whereas for BM capped Q-dots the absorption shows two band maxima at around two Gaussian functions (Fig. 3)^7^. The higher energy absorption band at 372 nm (G2) is assigned to the strongly quantized CdS clusters whereas the lower energy absorption band is at 427 nm (G1) might be corresponding to the aggregation. The first-derivative of the absorption spectrum or the Gaussian functions is also shown in Figs 1, 2, 3a. The absorption spectra of capped CdS Q-dots doped in a PMMA film are nearly the same as obtained in solution^8^.

Application of electric field to the CdS Q-dots doped in a rigid PMMA film perturbed the electronic energy states of the Q-dots, as a result a shift in the energy to lower and higher sites occurs, i.e., the absorption spectrum is shifted to lower and higher energy sites. The magnitude of the shift is determined by changes in the electric dipole moment (*μ*) and polarizability (*α*) of the electronic state involved.

Figures 1, 2, 3(b) show the E-A spectra of capped CdS quantum dots measured with the field strength of 0.7 MV cm^−1^. The polarized angle dependence E-A spectra were recorded with the incident angle normal to the surface (χ = 90^0^) and with the magic angle (χ = 54.7^0^) under atmospheric conditions at 2ω of the applied electric field (***F***). At both the angles, the E-A spectra are nearly the same, as shown in Figs 1, 2, 3(b). The E-A spectra crossing to the zero intensity line at same point (as shown in the overlapped spectra), indicating an isotropic distribution and immobility of CdS Q-dots in a PMMA film even in the presence of external electric field, and nearly zero ground state dipole moment. The amplitude of ΔA is proportional to the square of the applied electric field strength, i.e., quadratic in nature. Each of the negative lobes of the E-A spectra is representing to the transition from ground to the first exciton states. The negative lobes of the E-A spectra are approximately at the same position to the absorption maxima, or Gaussian functions confirming the spectral broadening as the basis for the observed electric field effects. Thus, the E-A spectra follow exactly to the second-derivative of absorption spectrum or the sum of the second-derivative of Gaussian functions used to reproduced the absorption spectrum, indicating that the E-A signals i.e., the field-induced change in absorption intensity mainly comes from the change in electric dipole moment (*μ*), that is (*Δμ*) following the transition to the first exciton state. For all the three types of capped Q-dots, the E-A spectra around first exciton band region could be fitted well by the second-derivative of the corresponding absorption, or the second-derivative of the Gaussian function used to reproduce the absorption spectrum, as shown in Figs 1, 2, 3(c). To reproduce the observed E-A spectra, both the zeroth and first derivatives of absorption spectrum are not necessary to be considered, i.e., the coefficients of *A*_*χ*_ and *B*_*χ*_ are negligible for these capped Q-dots, indicating that the transition dipole moment and polarizability to the first exciton state is hardly influence by ***F***. Actually, the observed E-A spectra for all type of capped Q-dots could be reproduced fairly well with the second-derivative of the corresponding absorption spectrum, and/or the Gaussian functions used to reproduced the absorption spectrum. Accordingly, a large change in permanent dipole moment 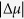 associated with the optical transitions from the ground to exciton states were evaluated following equations 1, 4 and 6 (Table 1). Note that change in the dipole moment i.e., 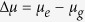 can have both negative and positive values depending on the value of (*μ*_*g*_) and (*μ*_*e*_). If (*μ*_*g*_) is smaller than the (*μ*_*e*_), then *Δμ* will be positive, i.e., enhancement in the dipole moment. On the other hand, if (*μ*_*g*_) is larger than the (*μ*_*e*_) then *Δμ* will be negative, which is unusual and truly unexpected. As shown in Table 1, all the estimated values are positive. The BT and BM capped CdS Q-dots exhibit a large value of 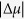 following the transition to the first exciton state. The positive value of 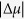 indicates that the electric dipole moment *μ* of the CdS Q-dots is enhanced in the first exciton state with respect to the ground state. Thus, the CdS Q-dots have large charge-transfer (CT) character in the first exciton state. The strong CT character may be due to the higher number of charge carrier on or near the nanocrystallite surface. The results indicate that the size of the Q-dots enhanced the CT character in the exciton state.

**Figure 4:**
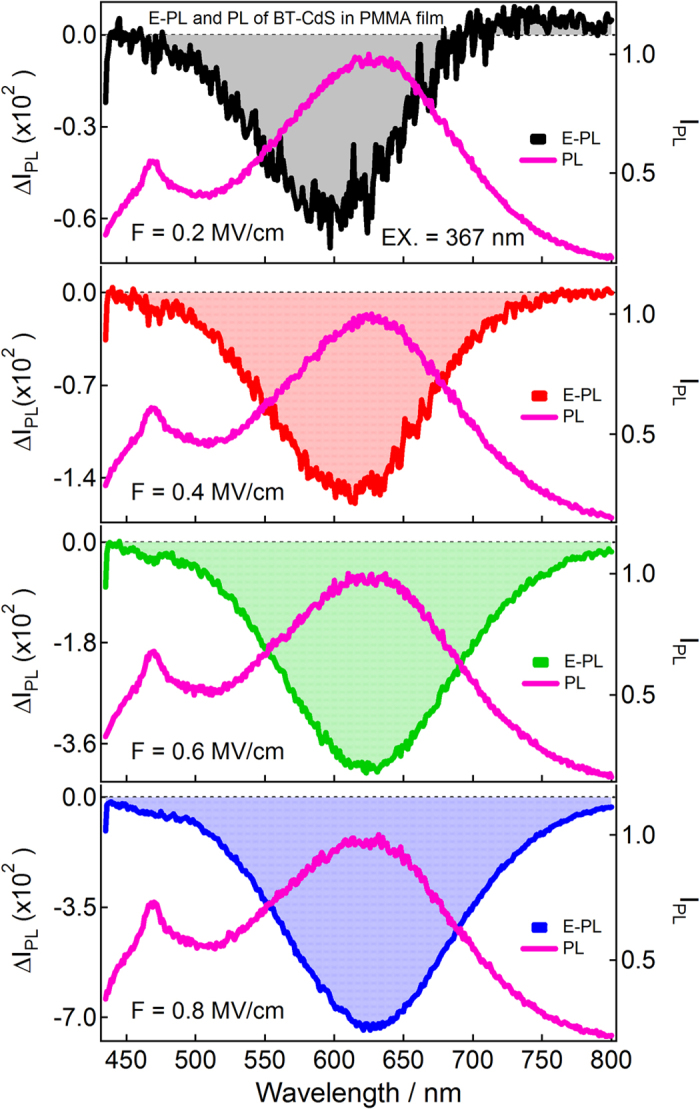
Electro-photoluminescence (E-PL) and photoluminescence (PL) spectra of BT-capped CdS Q-dots embedded in a PMMA film obtained with field strength of 0.2–0.8 MV cm^−1^ in vacuum near 295 K. Excitation wavelength was 367 nm.

**Figure 5:**
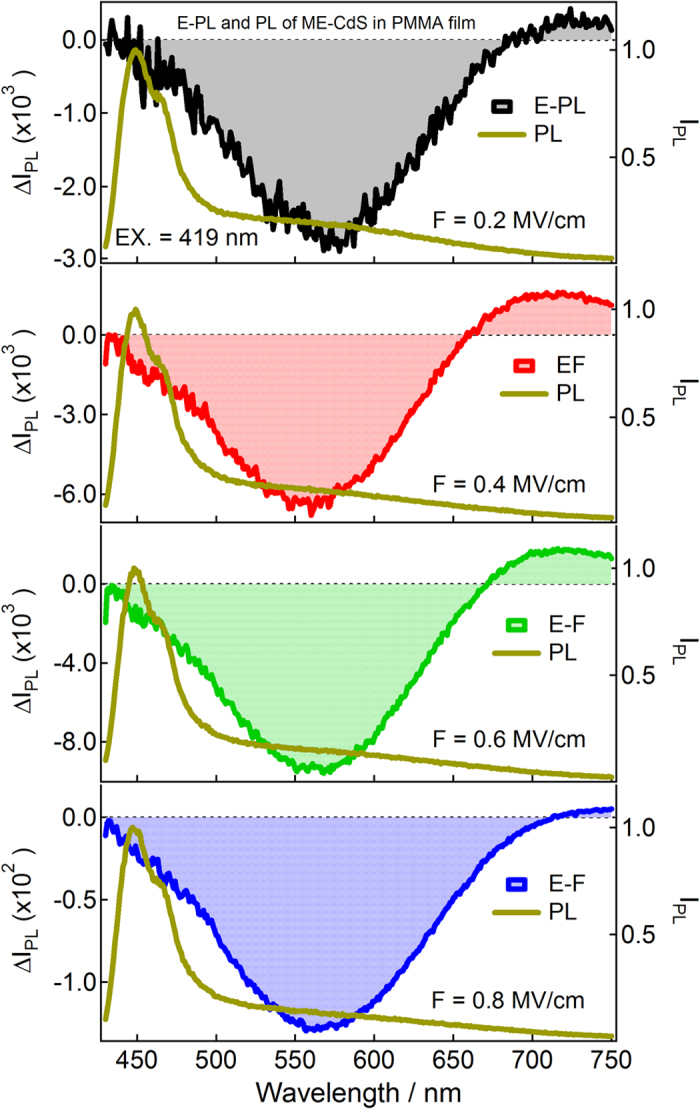
Electro-photoluminescence (E-PL) and photoluminescence (PL) spectra of ME-capped CdS Q-dots embedded in a PMMA film obtained with field strength of 0.2–0.8 MV cm^−1^ in vacuum near 295 K. Excitation wavelength was 419 nm.

**Figure 6:**
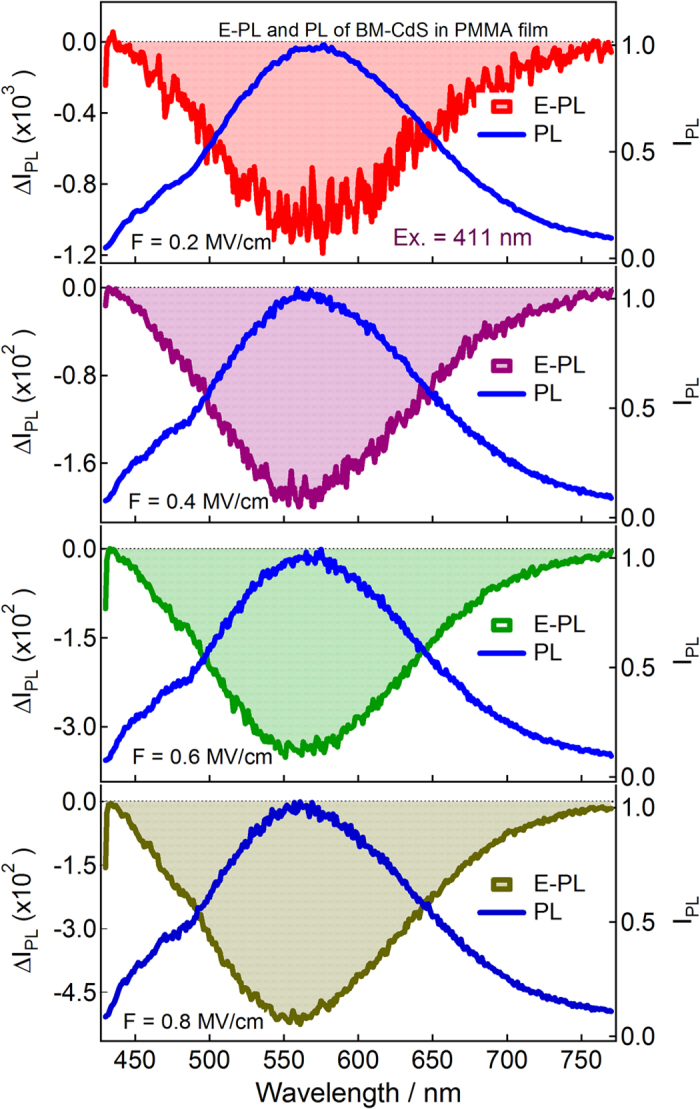
Electro-photoluminescence (E-PL) and photoluminescence (PL) spectra of BM-capped CdS Q-dots embedded in a PMMA film obtained with field strength of 0.2–0.8 MV cm^−1^ in vacuum near 295 K. Excitation wavelength was 411 nm.

Figures 4, 5, 6 show photoluminescence and electro-photoluminescence spectra of capped CdS Q-dots doped in a PMMA films. In all cases, the PL spectrum shows a broad band in the range of 420–800 nm. The position of the band maximum is different for different Q-dots; this is because of the distinct shape and size of Q-dots. The band maxima are at around 626 nm for BT, 566 nm for ME and 564 nm for BM capped CdS Q-dots. The large spectral width of the observed PL band is caused by inhomogeneous broadening due to a variation in particle size and broadening due to electron-phonon coupling^23^, whereas the large Stoke’s shift indicates that the emitting state is not the same as the optically excited state. In case of ME capped CdS Q-dots doped in a PMMA film, a comparatively smaller PL intensity was observed and the band is also dominated by a typical band appeared at shorter wavelength side in the range of 400–500 nm, the intensity of such a spurious band is very small when the PL spectra measured in solution^8^ however, we are unable to comment exactly on it at present.

It is evident that the shape, band maximum and the intensity of photoluminescence spectrum observed in vacuum and in ambient air conditions remain same, demonstrating that the organized capped CdS Q-dots are quite stable with respect to the environments. It is also evident that the spectral intensity of without capped CdS Q-dots, which shows two strong emission bands (particle size ≥ 2.9 nm) largely depends on the atmospheric conditions, and the observed photoluminescence spectra are different in vacuum condition and in atmospheric condition^5^. A large amount of intensity fluctuation was observed in PL when measured in vacuum with respect to the atmospheric conditions. The effect of environment was critically examined and explained^5^. The absorption and PL spectra observed for CdS Q-dots doped in a PMMA film are nearly same in shape to that of the observed in solvents, except there is a small shift in PL peak positions^8^, which may be due to the host polymer film. The CdS nanoparticles are extensively known for the two emission bands in two different regions. These bands are in the range of 400–520 nm and 520–800 nm^5^,^6^,^7^,^8^,^9^,^10^,^11^,^12^. The higher and lower energy bands are attributed to a direct recombination of *electron-hole* at the band gap and to the transition from surface atoms to the ground state, respectively. A single emission band was also observed in between 525–610 nm^24^.

Electro-photoluminescence spectra of capped CdS Q-dots doped in a PMMA film were measured under vacuum condition at 295 K with a varying field strength of 0.2–0.8 MV cm^−1^, as illustrated in Figs 4, 5, 6. PL and E-PL spectra were measured simultaneously, and the excitation were done at the selective wavelengths at which the E-A signal was negligibly small. PL spectra recorded at different excitation wavelengths are essentially the same; however, field-induced change in PL intensity varies with the wavelength of excitation^7^ as shown in supplementary information Figs S1 and S2.

The E-PL spectra (shown by shaded line) of BT, ME and BM capped CdS Q-dots measured at the field strength of 0.2 MV cm^−1^ are nearly identical to the negative of the PL spectrum (solid line), as shown in Figs 4, 5, 6. The integrated intensity of the entire E-PL spectrum is negative, even at low field of 0.2 MV cm^−1^. This clearly indicates that the photoluminescence quantum yield (QY) decreases in the presence of applied electric field. Thus, the low applied electric field strength of 0.2 MV cm^−1^ diminished the PL intensity of capped CdS Q-dots. The magnitude of the PL quenching monotonically increases with increasing field strength from 0.2–0.8 MV cm^−1^, as shown in Figs 4, 5, 6. The fractional field-induced quenching of PL of BT, ME and BM capped Q-dots determined by integrating the ratio of ΔI_PL_ to I_PL_ is 7.8 ± 0.2%, 9.6 ± 0.2% and 4.5 ± 0.2%, respectively at the applied field strength of 0.8 MV cm^-1^, without applying the lock-in amplifier correction factor (2√2). Here, I_PL_ and ΔI_PL_ represent the PL intensity at zero fields and the field-induced change in PL intensity respectively. The field strength dependence plots, i.e., ΔI_PL_/I_PL_ obtained by integrating the E-PL and PL spectra over the entire spectral region of 420–770 nm is nearly linear with the square of the electric field (*F*^2^). Thus, the field-induced quenching of PL strongly depends on the strength of the applied electric field. It should be pointed out that in each case the E-PL with respect to PL shows a red shift at lower values of electric field, i.e., the Stark shift. With increasing electric field strength, the magnitude of the shift becomes smaller (Figs 4, 5, 6), indicating that the field-induced quenching in PL becomes more significant than the Stark shift. It is important to note that the magnitude of the quenching depends on the capping ligands used to control the shape and size of Q-dots. It is noticed that the PL observed in the 400–500 nm region does not shows any considerable field effects even for ME capped Q-dots for which the higher energy band is very strong. No significant field-induced quenching or Stark shifts. This implies that the PL originated at around 450 nm for ME-capped Q-dots cannot be assigned to the direct recombination of *e-h* pair at the band gap, not to the vibronic or Raman band. On the other hand, the PL which arises at around 450 nm from the direct band gap of the CdS Q-dots influenced significantly by the application of electric field, as observed for the uncapped Q-dots^5^.

**Figure 7:**
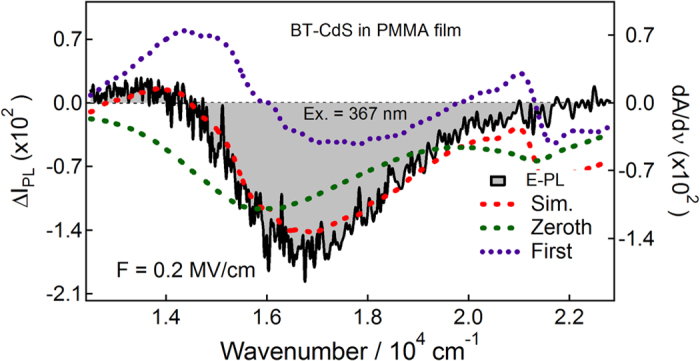
E-PL spectrum (shaded black line) of BT-capped CdS Q-dots embedded in a PMMA film observed with excitation at 367 nm with field strength of 0.2 MV cm^-1^. The simulated curve (dotted red line) and contribution of the zeroth and first-derivative (dotted lines) of PL spectrum used to reproduce the E-PL spectrum.

**Figure 8:**
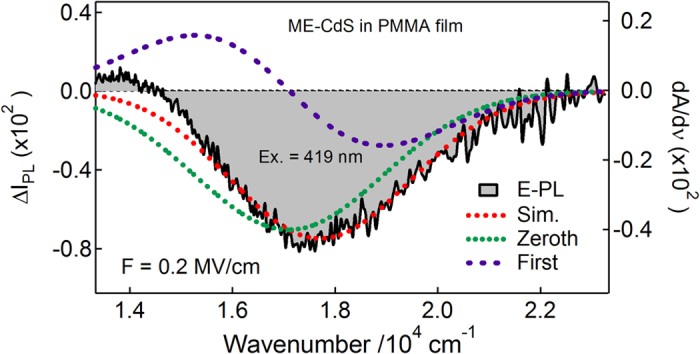
E-PL spectrum (shaded black line) of ME-capped CdS Q-dots embedded in a PMMA film observed with excitation at 419 nm with a field strength of 0.2 MV cm^−1^. The simulated curve (dotted red line) and contribution of the zeroth and first derivative (dotted lines) of PL (using Gaussian function) spectrum used to reproduce the E-PL spectrum.

**Figure 9:**
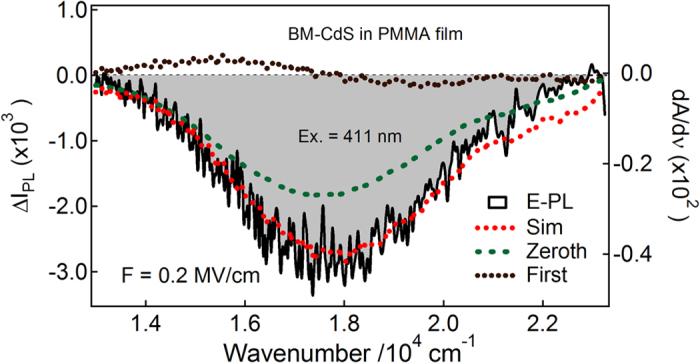
E-PL spectrum (shaded black line) of BM-capped CdS Q-dots embedded in a PMMA film observed with excitation at 411 nm with field strength of 0.2 MV cm^−1^. The simulated curve (dotted red line) and contribution of zeroth and first-derivative (dotted lines) of PL spectrum used to reproduce the E-PL spectrum.

To evaluate Stark shifts, the E-PL spectra of BT, ME and BM capped Q-dots were analyzed with the expression described by equations 2, 3, 4, 5, 6. In each case, the E-PL spectrum was reproduced by a linear combination of the zeroth- and first-derivative of the PL spectrum, the coefficient of the second-derivative spectrum is not necessary to be considered i.e., the contribution of second-derivative is negligible, as shown in Figs 7, 8, 9. The zeroth-derivative contribution measured the magnitude of field-induced quenching at 0.2 MV cm^−1^. However, the contribution of the first-derivative probably come from the orientation polarizability at room temperature because the emitting state has large electric dipole moment, and has been evaluated using the relation:
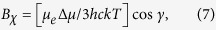
where *μ*_*e*_ represents electric dipole moment at the emitting state and Δμ represents difference in the electric dipole moment between ground and emitting state, respectively. *γ* is the angle between the vectors of *μ*_*e*_ and Δμ. Considering the negligible ground state dipole moment the change in dipole moment between the emitting state to the ground state has been evaluated and presented in table 1. Note that the E-A spectra are purely the second-derivative of the absorption, i.e. there is no contribution of the polarizability or orientational polarizability, which is in agreement of the E-PL spectra. As shown in Table 1, there is a large difference in the contribution of 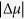 and field-induced quenching obtained for ME as comparison to the BT and BM capped Q-dots. This could be explained by the quantum confinement effects, the PL which is originated from the surface state instead of direct recombination of *e-h* pair at the band gap and the capping effects. In all cases, the PL originated from the direct recombination of *e-h* pair is absent or negligibly small. This fact is also applicable to understand the difference in 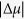 obtained by E-A and E-PL for capped Q-dots. The field-induced spectral broadening of E-A spectra originated due to enhancement of electric dipole moment in the excited state following the absorption, indicating CT character of Q-dots. Thus, both the E-A and E-PL spectra show the CT character of the Q-dots. The emitting state which is produced by the recombination of electron-hole is then dissociated into a carrier of hole and electron (charge-transfer state) in the presence of external electric field. The dissociation of *e-h* is also supported by the decrease in the initial population of emitting state^5^,^7^. In case of the ME capped Q-dots, the field-induced quenching is higher as compared to the BT and BM capped Q-dots indicating that the selection of the capping ligands are important to control the shape, size, stability and defects of Q-dots. Thus, understanding the CT character and field-induced quenching in Q-dots is equally important for using these Q-dots in LEDs, photovoltaic and biological applications. The field-induced quenching is important for the application of LEDs because electric field is always applied during the operation of LEDs.

## Conclusions

The E-A spectra of BT, ME and BM capped CdS Q-dots doped in a PMMA film were exactly same to the second-derivative of the absorption spectrum or the second derivative of the Gaussian functions used to reproduced absorption spectrum, demonstrating an enhancement of ***μ*** in the first exciton state following absorption. The E-A and E-PL spectra and the enhancement in ***μ*** in the exciton state indicates that the capped CdS Q-dots have larger charge-transfer character in the first exciton state. The photoluminescence intensity of uncapped CdS nanoparticles was strongly depends on the atmospheric conditions and shows intensity fluctuation with respect to air and vacuum^5^. Unlike to the uncapped Q-dots, capped CdS Q-dots shows a stable large Stoke’s sifted PL band originated from the surface state, the PL from direct band gap is trivially small or absent. The PL of capped CdS Q-dots is quenched significantly in the presence of applied electric field. At lower electric field, E-PL spectra are dominated mainly by the Stark shifts, whereas at higher electric field the E-PL spectra dominated by field-induced PL quenching, which is nearly linear with the square of applied electric field. The field-induced PL quenching and CT character are different for BT, ME and BM capped Q-dots. Thus, understanding the CT character and field-induced PL quenching is important for the application of Q-dots in LEDs, solar cell and biological applications.

## Methods

Thiol capped cadmium sulfide quantum dots/nanocrystallites were prepared with microwave (MW) irradiation method. 1-Butanethiol (BT), 2-mercaptoethanol (ME) and benzyl mercaptan (BM) were used as capping ligands. The detail procedure of synthesis of capped CdS Q-dots was discussed elsewhere^6^,^7^,^8^. The high resolution transmission electron microscopy (TEM) and FESEM^7^,^8^ images of BT, ME and BM capped CdS quantum dots are given in supplementary information (Fig S3). The sizes of the particles are estimated from absorption spectra following the method described in supplementary information (particle size estimation and Fig S4).

To prepare the capped Q-dots doped PMMA film, a certain amount of capped CdS Q-dots and PMMA was dissolved in chloroform and then deposited on ITO-coated quartz substrate using a spin-coating technique. The thickness of the CdS doped PMMA film, measured with an interferometric microscope was typically 0.5 μm. For the electric field measurements, a semitransparent aluminum (Al) film was further deposited on the PMMA film with a vacuum vapor deposition technique. The pre-coated ITO and post-coated Al films were used as electrodes. A sinusoidal ac voltage with a modulation frequency of 40 Hz was used for the measurements of electroabsorption (E-A) and electro-photoluminescence (E-PL) spectra.

The sample film was used for the measurement of absorption spectra with Hitachi U-3500 spectrophotometer and PL spectra with JASCO FP-777 spectrofluorometer. In the E-A measurement, the light beam from xenon lamp of JASCO FP-777 spectrofluorometer was collimated with a lens and passed through α-barium borate polarization prism (α-BBPP) and through the sample film on an external photomultiplier (PMT). A rotatory stage of α-BBPP was used for varying the angle between the polarization direction of the excitation light and the direction of the applied electric field. A signal from the PMT is then send to lock-in-amplifier and computer (PC). E-A and E-PL signals were recorded at the 2ω of the modulation frequency of the applied electric field, E-PL spectra were measured at room temperature with a non-polarized light under vacuum conditions. Detailed of the measurement procedure for E-A and E-PL spectra in presence and absence of external electric field were described elsewhere^14^,^15^.

## Additional Information

**How to cite this article**: Mehata, M. S. Enhancement of Charge Transfer and Quenching of Photoluminescence of Capped CdS Quantum Dots. *Sci. Rep.* **5**, 12056; doi: 10.1038/srep12056 (2015).

## Supplementary Material

Supplementary Information

## Figures and Tables

**Table 1:** Magnitude of change in dipole moment 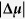 (D/*f*) and orientational polarizability 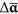
(Å^3^/*f*^*2*^) of CdS Q-dots obtained from electroabsorption (E-A) and electro-photoluminescence (E-PL) spectra.

**Capping ligands**	**Average particle size (nm)**	E-A 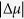 in Debye	E-PL 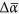 (Å)^3^	E-PL 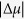 in Debye
BT	3.8 ± 0.2	25 ± 1.0	17000	22 ± 2.0
ME	4.0 ± 0.2	11 ± 0.5	26800	27 ± 2.0
BM	2.1 ± 0.2	29 ± 1.5 (G1)^a^	5400	12 ± 1.0
		20 ± 1.0 (G2)^a^	—	—
No ligand^b^	1.0	12 ± 1.0	—	—
	2.9	20 ± 1.0	—	—
	5.0	23 ± 1.0	—	—

^a^Ref. [7].

^b^Ref. [5].
