# Supplementary material for: Enhancement of Charge Transfer and Quenching of Photoluminescence of Capped CdS Quantum Dots
Source: Sci Rep. 2015 Jul 13;5:12056. doi: 10.1038/srep12056 (PMC4499802; doi:10.1038/srep12056)
Supplement: Supplementary Information [file srep12056-s1.docx]

**Supplementary Information**

**Enhancement of Charge Transfer and Quenching of Photoluminescence of Capped CdS Quantum Dots**

Mohan Singh Mehata

Laser-Spectroscopy Laboratory, Department of Applied Physics, Delhi Technological University, Bawana Road, Delhi 110042, INDIA

Corresponding address: [msmehata@gmail.com](mailto:msmehata@gmail.com); mohan.phy@dce.edu


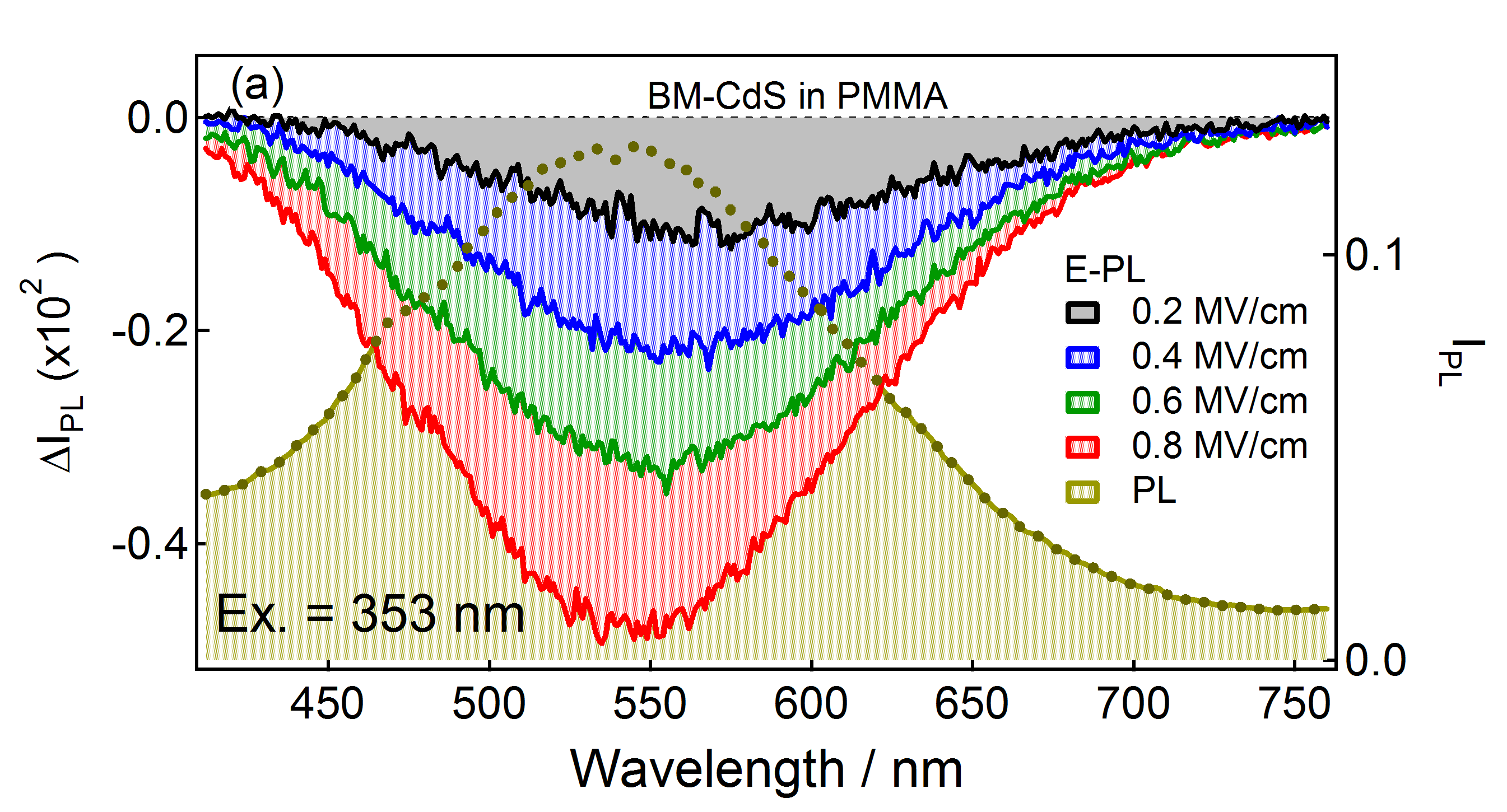


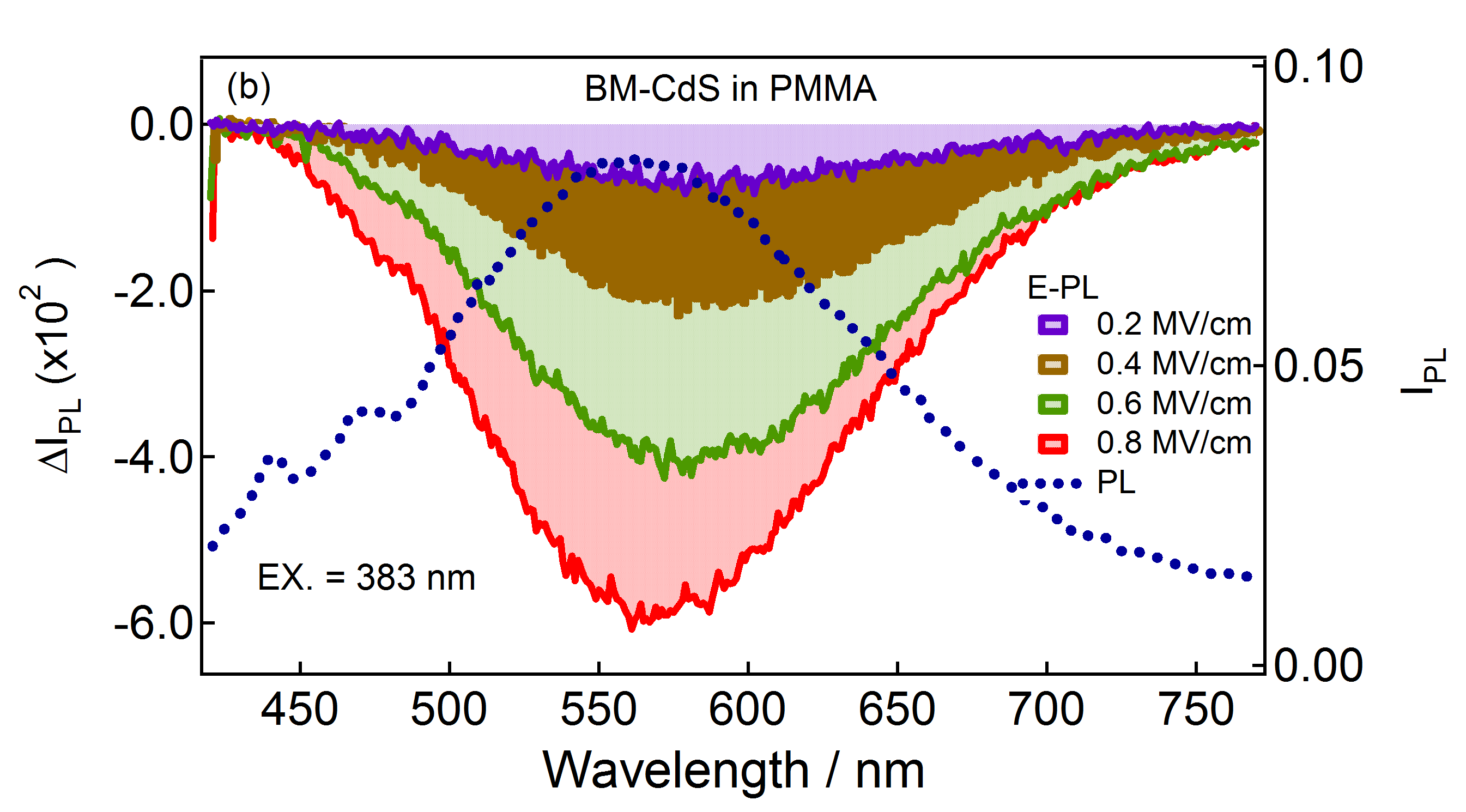


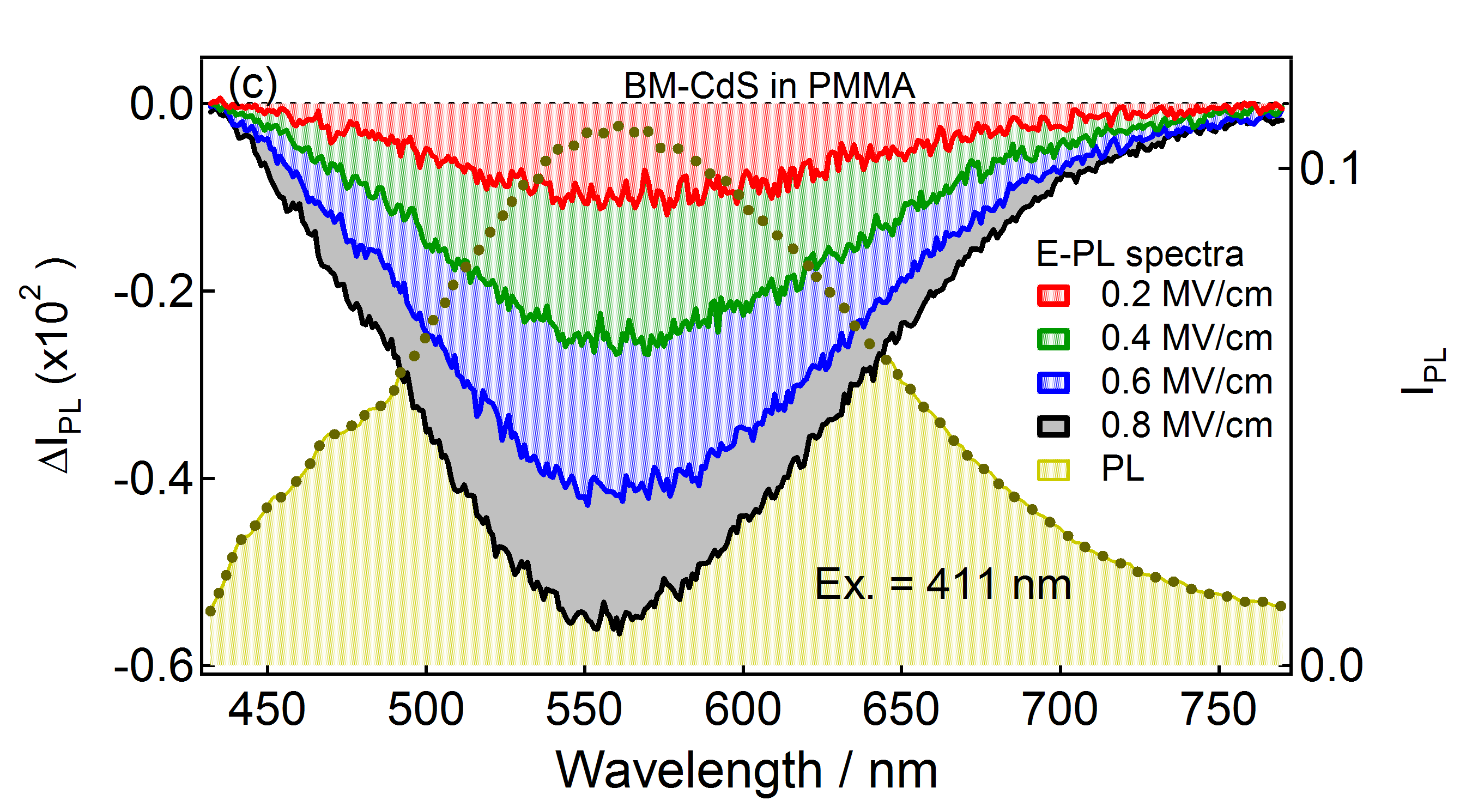


**Figure S1:** Electro-photoluminescence (E-PL) and photoluminescence (PL) spectra of BM-capped CdS Q-dots embedded in a PMMA film obtained with field strength of 0.2 - 0.8 MV cm^-1^ at different excitation wavelengths (a-c). The field-induced PL quenching of CdS Q-dots doped in PMMA film depends on electric field strength and excitation wavelengths.


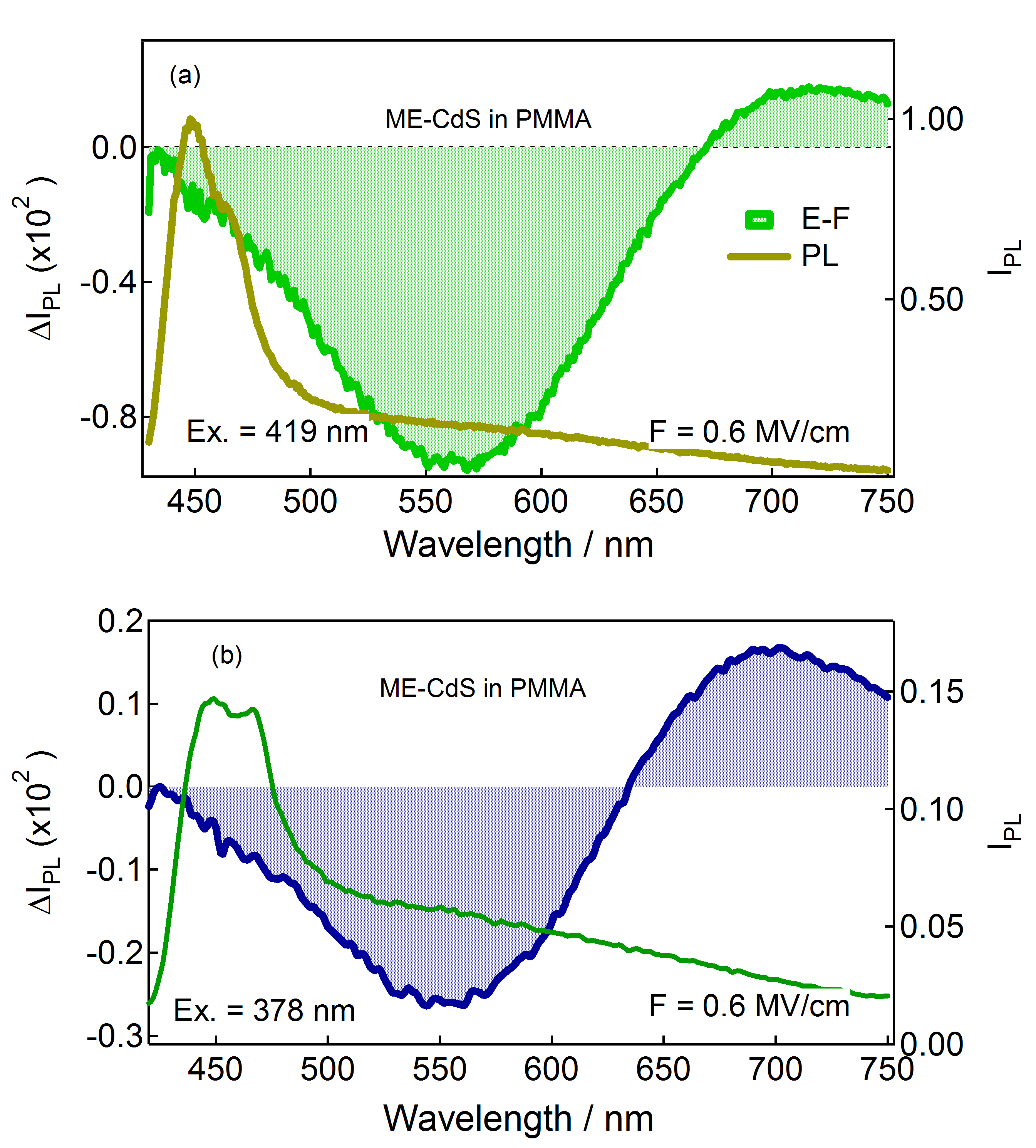


**Figure S2:** Electro-photoluminescence (E-PL) and photoluminescence (PL) spectra of ME-capped CdS Q-dots embedded in a PMMA film obtained with a field strength of 0.6 MV cm^-1^ at different excitation wavelengths. The field-induced PL quenching of CdS Q-dots doped in PMMA film depends on electric field strength and excitation wavelengths.


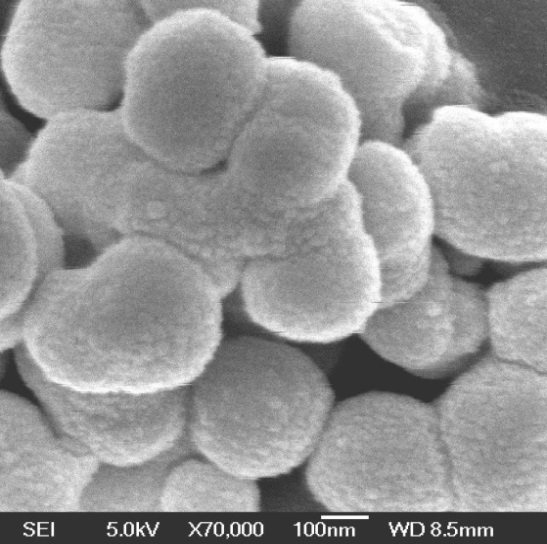


**(b)**


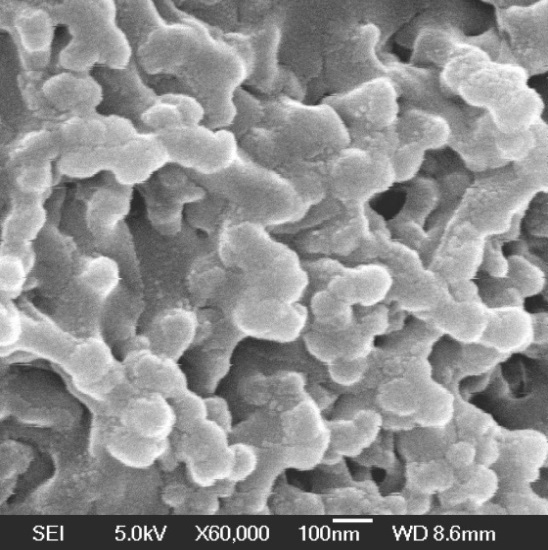


**(a)**


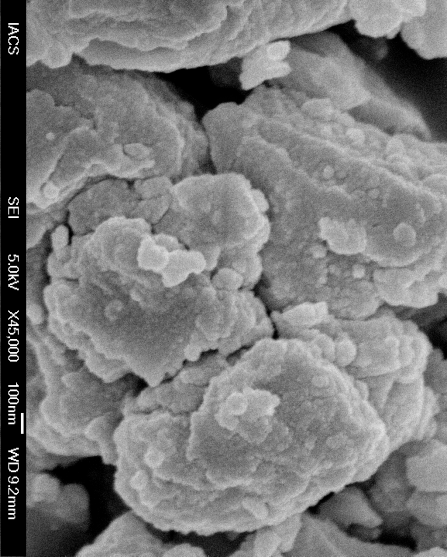


**(c)**


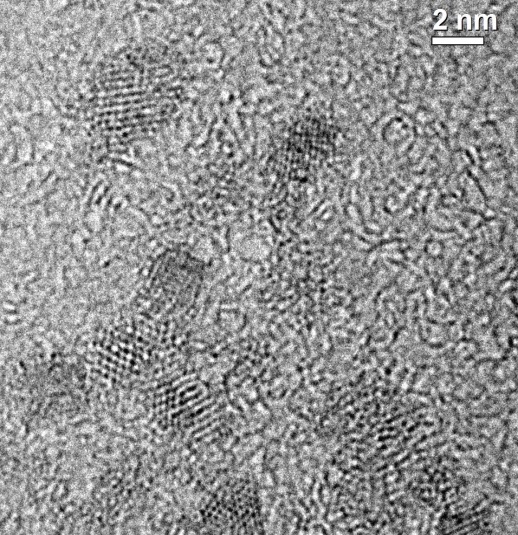


**(b)**


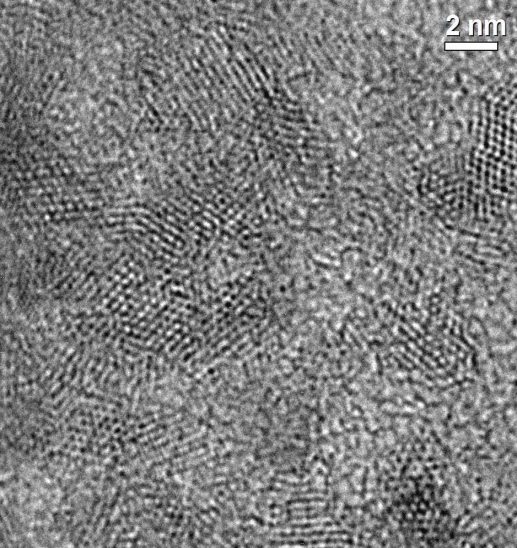


**(a)**


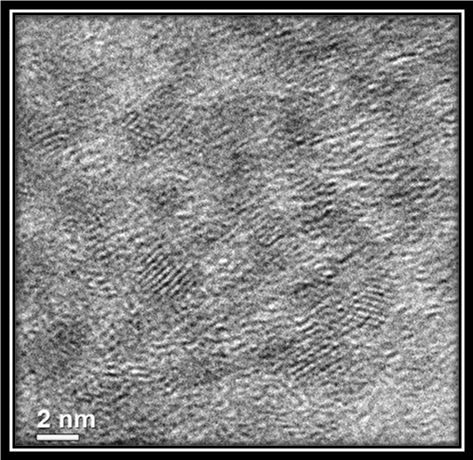


**(c)**

**Figure S3:**  High resolution transmission electron microscopy (HRTEM) (left) and FESEM (right) images of BT (a), ME (b) and BM (c) capped CdS Q-dots^1,2^.

**Particle Size Estimation**

The average size of capped CdS Q-dots was estimated from the obtained absorption spectra using the effective mass approximation^3^. The strong-confinement regime of the first excited electronic state can be approximated by the following equation:

$E_{g}= E_{g}^{bulk}+ \frac{h^{2}}{8 R^{2}}\left( \frac{1}{m_{e}^{*}}+ \frac{1}{m_{h}^{*}} \right)- \frac{1.8e^{2}}{4\pi\varepsilon_{0}\varepsilon R}$ , (1)

in which E_g_ is the optical transition energy of CdS Q-dots; $E_{g}^{bulk}$ is the band gap of the bulk CdS ($E_{g}^{bulk}$ = 2.4 eV); h is the Plank’s constant, R is the radius of the particle, $m_{e}^{*}$ (0.19 $m_{0}$) , $m_{h}^{*}$ (0.8 $m_{0}$) are the effective mass of electrons and hole in CdS, respectively^2^; $m_{0}$ is the electron rest mass, *ε_0_* is the vacuum permittivity and *ε* is the relative permittivity of CdS (ε = 5.7) for CdS^4,5^.

The energy band gap (E_g_) of CdS Q-dots was estimated from the absorption spectrum using Tauc’s relation, as given:

$\alpha h\vartheta=A{(h\vartheta-E_{g})}^{n}$, (2)

where *α* is the absorption coefficient, *hυ* is the photon energy, *A* is a constant and *n* assumes the values 1/2, 2, 3/2 and 3 for allowed direct, indirect, forbidden direct and indirect transitions, respectively. For allowed direct transitions the relation can be given as:

$\alpha h\vartheta=A{(h\vartheta-E_{g})}^{1/2}$ (3) Then the plots between (αhυ) ^2^ *vs* (hυ) for different CdS Q-dots are obtained (Fig. S4). The E_g_ determined by extrapolation of the linear regions on the energy axis (Fig. S4) is 2.8, 2.79 and 4.2 eV for BT, ME and BM CdS Q-dots, respectively. Then from equation (1) the estimated size of BT, ME and BM CdS Q-dots comes out to be 3.8, 4.0 and 2.1 nm for BT, ME and BM capped CdS Q-dots, respectively.










**Figure S4:**  Tauc’s plots obtained between (αhυ)^2^ and (hυ) for (a) BT, (b) ME and (c) BM capped CdS Q-dots.

**References:**

1. Mehata, M. S., Majumder, M., Mallik, B. & Ohta, N. External electric field effects on optical property and excitation dynamics of capped CdS quantum dots embedded in a polymer film*. J. Phys. Chem. C* **114**, 15594–15601 (2010).
2. Majumder, M., Karan, S. & Mallik, B. Study of steady state and time resolved photoluminescence of thiol capped CdS nanocrystalline powders dispersed in N,N-dimethylformamide. *J. Lumin.* 131, 2792–2802 (2011).
3. Brus, L. E. Electron-electron and electron-hole interactions in small semiconductor crystallites: The size dependence of the lowest excited electronic state. J. Chm, Phys. **80**, 4403-4409 (1984).
4. Lippens, P. E & Lannoo, M. Calculation of the band gap for small CdS and ZnS crystallites. Phys. Rev. B **39**, 10935 (1989).
5. [Tamiolakis](http://pubs.rsc.org/en/results?searchtext=Author%3AIoannis%20Tamiolakis), I.,   [Lykakis](http://pubs.rsc.org/en/results?searchtext=Author%3AIoannis%20N.%20Lykakis), I. N, [Katsoulidis](http://pubs.rsc.org/en/results?searchtext=Author%3AAlexandros%20P.%20Katsoulidis), A. P., & [Armatas](http://pubs.rsc.org/en/results?searchtext=Author%3AGerasimos%20S.%20Armatas), G. S. One-pot synthesis of highly crystalline mesoporous TiO_2_ nanoparticle assemblies with enhanced photocatalytic activity***. Chem. Commun.*, 48**, 6687-6689 (2012).
